# *Escherichia coli*: Physiological Clues Which Turn On the Synthesis of Antimicrobial Molecules

**DOI:** 10.3390/vetsci7040184

**Published:** 2020-11-21

**Authors:** Sarah-Jo Paquette, Tim Reuter

**Affiliations:** 1 University of Lethbridge, Lethbridge, AB T1K 3M4, Canada; sarahjo.paquette@alumni.uleth.ca; 2 Alberta Agriculture and Forestry, Lethbridge, AB T1J 4V6, Canada

**Keywords:** *Escherichia coli*, pathogen, antimicrobial molecules, inhibition, physiological cues, antimicrobial production, microcins, therapeutic

## Abstract

Zoonotic pathogens, like Shiga toxin-producing *Escherichia coli* (STEC) are a food safety and health risk. To battle the increasing emergence of virulent microbes, novel mitigation strategies are needed. One strategy being considered to combat pathogens is antimicrobial compounds produced by microbes, coined microcins. However, effectors for microcin production are poorly understood, particularly in the context of complex physiological responses along the gastro-intestinal tract (GIT). Previously, we identified an *E. coli* competitor capable of producing a strong diffusible antimicrobial with microcin-associated characteristics. Our objective was to examine how molecule production of this competitor is affected by physiological properties associated with the GIT, namely the effects of carbon source, bile salt concentration and growth phase. Using previously described liquid- and agar-based assays determined that carbon sources do not affect antimicrobial production of *E. coli* O103F. However, bile salt concentrations affected production significantly, suggesting that *E. coli* O103F uses cues along the GIT to modulate the expression of antimicrobial production. Furthermore, *E. coli* O103F produces the molecule during the exponential phase, contrary to most microcins identified to date. The results underscored the importance of experimental design to identify producers of antimicrobials. To detect antimicrobials, conventional microbiological methods can be a starting point, but not the gold standard.

## 1. Introduction

Physiological adaptations are defined as ‘a metabolic or physiologic adjustment within the cell, or tissues, of an organism in response to an environmental stimulus resulting in an altered ability of that organism to cope with its changing environment’ [1]. These adaptations affect all bacterial systems from defense to growth and are reactions to their fluctuating ecosystems to enable survival [2]. When bacteria encounter changes in their surroundings, modifications in gene expression are triggered for a physiological adaptive response to these variations [3].

*Escherichia coli* (*E. coli*) is a Gram-negative, facultative anaerobe, which typically resides in the gastro-intestinal tract of mammals [4,5]. However, *E. coli* is viable outside mammalian hosts and inhabits various environments such as soil and water [5,6,7]. In fact, a comparison of genomes from environmental and enteric *E. coli* identified genes that were unique to each environment, such as production of diol utilization for energy (environmental) and transport of nutrients typically associated with gut environments (enteric), further demonstrating the range of bacterial adaptability [7].

*E. coli* has a unique adaptation strategy to survive and persists in the gastrointestinal tract (GIT) of warm-blooded animals including humans. Ingested *E. coli* encounter several hurdles along the GIT and must overcome these obstacles to occupy the large intestine [4,6,8]. The first hurdle is the low pH of the stomach, which *E. coli* counteracts with sophisticated acid defense systems [8,9,10].

These defense systems are often accredited to the low infectivity dose of Shiga toxin-producing *E. coli* (STEC). After passing through the stomach, *E. coli,* as a fellow passenger of ingested food, is subjected to bile discharged into the small intestine [4]. Mainly facilitating the digestion of dietary fats, bile salts are bactericidal and thought to prevent bacterial overgrowth along the small intestine [4,11,12]. To counteract bile, *E. coli* adapted various mechanisms such as efflux-pumps. For food-borne pathogens such as STEC, the bile salt concentration has been shown to trigger a signal to down-regulate genes associated with the locus of enterocyte effacement (LEE) and up-regulate iron acquisition genes [13].

Another major hurdle for invasive *E. coli* is the competitive microbiome in the large intestine [4]. Newcomers compete with the existing microflora for space and nutrients, the focal point of competitive interactions [14]. One unique way how pathogenic *E. coli* out-compete commensals is through advanced carbohydrate utilization [15]. In contrast, *E. coli* commensals can counteract STEC by producing bacteriocins [16], coined colicins and microcins [17]. Bacteriocins are vital in mediating microbial populations [18] and under consideration as potential antimicrobial therapeutics [17].

Previously, by creating an agarose-based physical barrier between competitive *E. coli* strains, our so-called omelette method, we identified a champion strain (O103F) that produced one and/or a complex of strong diffusible molecule(s) (subsequently referred to as molecule) with antimicrobial properties that maintained a strong zone of no growth monitored for up to 7 days and had the strongest zone of no growth even when the agar barrier thickness and distance between competitors increased incrementally [19]. Subsequent experiments using a molecule extraction assay revealed that the molecule has microcin characteristics, including the persistence to physiochemical conditions (pH 3 and 11, autoclave temperature ≥121 ℃ and pressure ≥ 12.4 N/cm^2^) as well as enzymatic digestion treatments [20]. Based on our current understanding, no viable biological molecule can persist autoclave conditions with the exception of misfolded prions, endospores and microcins, suggesting that after micro filtration and subsequent treatment, the remaining molecule is likely a microcin. However, whether any molecule production is affected by physiological properties has not yet been fully discovered. Cameron et al., including the author TR of this study [21], identified unintentionally that changing media formulations with the absence of bile salts did not result in the detectable production of antimicrobial molecules, demonstrating that growth conditions can have a tremendous impact on the production of antimicrobial compounds.

Furthermore, studies with *E. coli* O157 reported the up/down-regulation of specific genes in response to: (I) physiological changes encountered along the digestive tract [9,13,22] or (II) the type of carbon nutrients [23].

The primary aim of this study was to use in vitro experiments mimicking characteristics within the GIT. Our objectives were to determine the effect of carbon source, bile salt concentrations and growth phase on the antimicrobial molecule production of our *E. coli* O103F champion. A second objective was to further investigate the molecule specificity to unveil whether the Enterobacteriaceae *Salmonella* or *Klebsiella* were also impaired by the *E. coli* O103F molecule.

## 2. Materials and Methods

### 2.1. Bacterial Strains: Cultures, Media and Culture Conditions

Both *E. coli* strains (O103F and O157A) used in this study were described previously [19]. The strain O103F was tested negative for virulence genes including stx1, stx2, hlyA and eae, while O157A (O157:H7) is an STEC tested positive for those virulence factors, respectively [19]. *E. coli* were streaked from glycerol stocks onto MAC. Plates were incubated overnight (O/N, 16–18 h) at 37 °C. A single colony was selected from each plate and inoculated into specific liquid media (Table 1) depending on the objectives (described below) and incubated O/N at 37 °C with shaking at 150 rpm. *Klebsiella pneumoniae* and *Salmonella typhimurium* isolates (Lethbridge reference library) were streaked from glycerol stocks onto TSA. Plates were incubated O/N (16–18 h) at 37 °C. A single colony was selected from each plate and inoculated into TSB incubated O/N at 37 °C with shaking at 150 rpm. Cell numbers were analysed by optical density (OD) measured at a wavelength of 600 nm. Cell number related OD data were validated against the CFU enumeration of plated serial dilution in the range of 30–300 CFU using conventional plate-counting at various timepoints over the duration of this study (Supplemental, Figure S1).

Culture conditions for the Molecule extraction assay [20]: O/N cultures of O157A and O103F were diluted to an OD of 0.1 measured at a wavelength of 600 nm in either fresh EC, LB, TSB, TSB/EC or TSB+ (Table 1) and grown for 3 h. The 3 h culture was then used as inoculum for molecule isolation assays.

Culture conditions for the Omelette method [19]: Testing different media for molecule production, O103F and O157A O/N cultures (EC) were used to set-up omelette agar plates using TSA, MAC, PN, MH, and LBA (Table 1). Testing molecule specificity, *E. coli* O103F, *Klebsiella* and *Salmonella* O/N cultures were used to set-up omelette plates using MAC agar only.

### 2.2. Effect of Carbon Source on Molecule Production

Liquid and solid media (Table 1 overview and Supplemental Table S1 with detailed media composition) were used to examine *E. coli* O103F molecule production to identify whether changing nutrients affected O103F ability to inhibit *E. coli* O157A growth.

(a) Molecule Extraction Assay:

Molecule extraction assays were performed as previously described [20]. In short, both O103F and O157A were grown separately for 12 h in liquid media and subsequently filter-sterilized to remove all the bacterial cells and complex biomolecules by size exclusion. The filtered supernatant was then used as inoculation media for O157A (inhibition assay). The supernatant recovered from O103F will subsequently be referred to as **A**nti**M**icrobial **Mo**lecule (AMMO) and the supernatant from O157A as SPENT (a control, to take into account the effect of depletion of nutrients and metabolic end products in the extracted supernatants on O157A growth). The inoculation media for the inhibition assay was composed of 75% SPENT or AMMO, and 25% was a mix of fresh media and bacterial cells (standardized to an OD_600nm_ of 0.1). Differences in growth between the supernatants was monitored by OD. To test the effect of different carbon sources, EC, TSB, LB or TSB+ (Table 1) were used as media.

(b) Omelette method:

The omelette method was performed as previously described [19]. In short, the O103F competitor was inoculated on one side of an agar plate and incubated for 24 h. After the incubation, the agar was flipped and O157A inoculated perpendicular on the other side of the agar. The plate was incubated another 24 h and monitored for the presence of zones of no growth. The bottom strain was always the *E. coli* O103F champion and the top strain was the *E. coli* O157A competitor. To test the effect of different carbon sources, MAC, LBA, TSA, PN or MH agar (Table 1 and Table S1) was used as media for the method.

### 2.3. Effect of Bile Salts on Molecule Production

The effect of bile salts was examined using the molecule isolation assay as previously described with TSB (no bile salts–0% bile salts), 50:50 mix of TSB and EC (0.75 g/L bile salts in liquid media—50% bile salts) and EC (1.5 g/L bile salts in liquid media—100% bile salts) as growth medium (Table 1).

### 2.4. Effect of E. coli O103F Growth Stage on Molecule Production

Molecule isolation assays were performed as previously described [20], with the following modification. To test the effect of *E. coli* O103F growth time on molecule production, a continuous culture of *E. coli* O103F in EC was grown, and at timepoint 4, 6, 8, 10 and 12 h, a subsample was taken and the OD_600nm_ was recorded, followed by molecule isolation and used as AMMO for the subsequent assays. An *E. coli* O157A SPENT control was also grown in EC, adjusted to match the OD_600nm_ value of *E. coli* O103F at each timepoint to balance nutrient depletion in the SPENT media control with that of the O103F AMMO media.

### 2.5. Specificity of the E. coli O103F Molecule

The previously described omelette method [19] was used to assess the specificity of the O103F antimicrobial against *Salmonella* or *Klebsiella* as the competitor. In short, the O103F competitor was inoculated on one side of a MAC agar plate and incubated for 24 h. After the incubation, the agar was flipped and either *Klebsiella* or *Salmonella* was inoculated perpendicular on the other side of the agar. The plate was incubated and monitored for presence of zones of no growth at timepoints 24, 48 and 72 h.

### 2.6. Statistical Analysis

Numerical OD data measured for the molecule isolation assays were examined for normality and subsequently used for analyses. Time, media, control, and interactions were determined for all the experiments using a mixed linear model (Proc Mixed, SAS 9.4, SAS Institute Inc., Cary, NC, USA). *p* values < 0.05 were considered significant.

## 3. Results

### 3.1. Effect of Carbon Source on Molecule Production

#### 3.1.1. Molecule Isolation Assay—Molecule Production in Different Liquid Media

**Figure 1:**
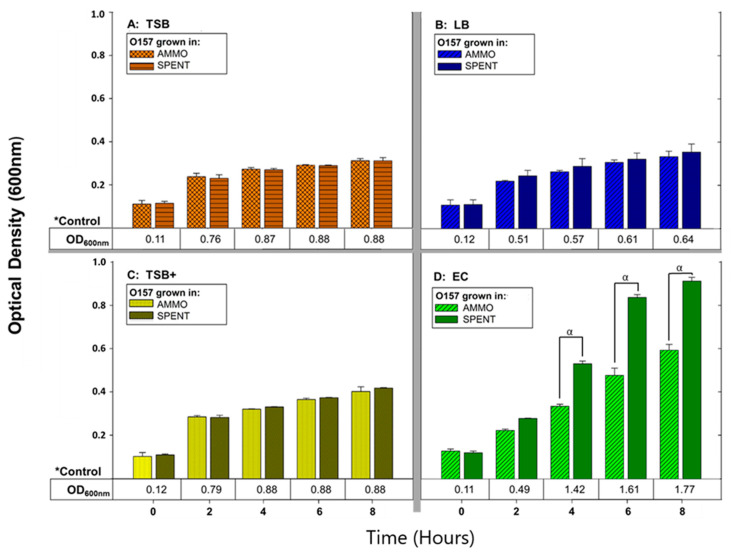
The effect of different media (TSB, LB, TSB+ and EC) on ability of AMMO to inhibit growth of *E. coli* O157A measured using OD_600nm_. O157A controls^(^*^)^ in fresh media are presented as a numerical value below the OD data. The symbol **α** denotes a significant difference between O157A grown in AMMO and in the SPENT for EC, (*p* < 0.05). Note: (1) AMMO is the cell-free supernatant collected after 12 h of *E. coli* O103F growth. SPENT is the cell-free supernatant collected after 12 h of *E. coli* O157A growth. (2) Bars are the calculated standard deviation for the treated or untreated AMMO and SPENT at each timepoint.

Comparison of O157A OD enumerations grown in AMMO (extracted from O103F grown 12 h in EC) to O157A grown in SPENT control (extracted from O157A grown 12 h in EC), demonstrated that O157A grown in AMMO was significantly different in growth at timepoints 4, 6 and 8 h (*p* < 0.05) compared to O157A grown in SPENT (Figure 1D). In contrast, comparison of OD enumerations for O157A grown in AMMO extracted from various media (TSB, LB and TSB+) to O157A grown in SPENT from the same media as AMMO were without significant differences in growth at timepoints 4, 6 and 8 h (*p* > 0.05) (Figure 1A–C). Predictably, controls of O157A grown in either, fresh TSB, LB, TSB+ and EC were recorded with a higher cell density than the corresponding SPENT control (Figure 1A–D, respectively), as SPENT was initially composed with 75% of a gradually depleted media as well as containing metabolic end products.

#### 3.1.2. Omelette Method—Molecule Production on Different Solid Media

**Figure 2:**
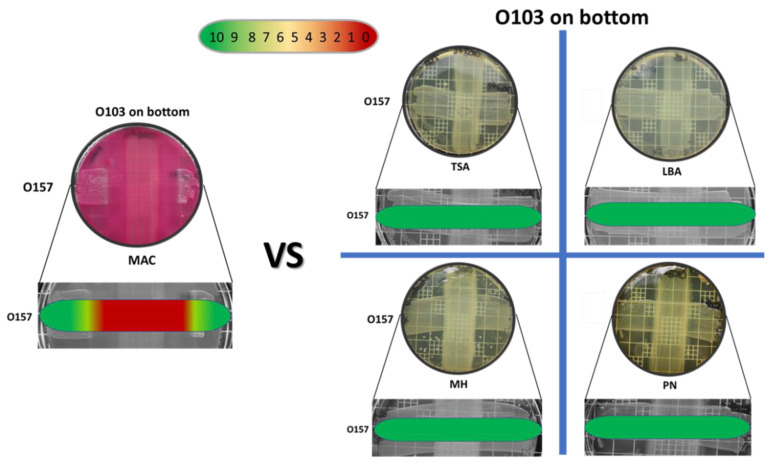
Omelette method results for *Escherichia coli* O103F against O157A on five different agars, MAC, TSA, LBA, MH and PN examining zones of no growth after an incubation of 24 h. Growth zones are graded beneath each plate with red (0) representing no growth to green (10) representing no inhibition of growth.

The semi-quantitative data examining the effect of O103F molecule production using different solid media (LBA, TSA, PN, MH and MAC) identified that molecule production only occurred on MAC agar, as seen by the strong zone of no growth against the O157A competitor at 24 h (Figure 2). On all other media tested, the O157A grew over the O103F competitor and O103F did not produce a zone of clearing (Figure 2).

### 3.2. Effect of Bile on Molecule Production

**Figure 3:**
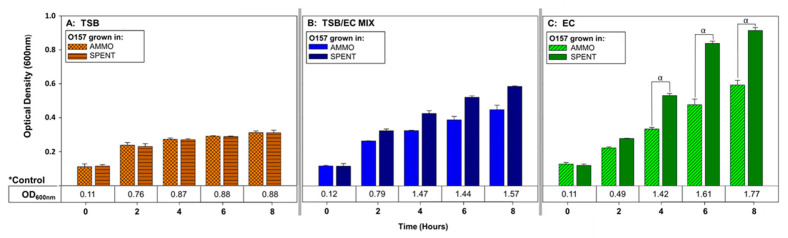
The effect of bile salts on ability of AMMO to inhibit growth of *E. coli* O157A measured using OD_600nm_ using TSB, TSB/EC mix and EC. O157A controls^(^*^)^ in fresh media are presented as a numerical value below the OD data. The symbol **α** denotes a significant difference between O157A grown in AMMO and in the SPENT for EC, (*p* < 0.05). Note: (1) AMMO is the cell-free supernatant collected after 12 h of *E. coli* O103F growth. SPENT is the cell-free supernatant collected after 12 h of *E. coli* O157A growth. (2) Bars are the calculated standard deviation for the treated or untreated AMMO and SPENT at each time point.

Comparison of OD enumerations of O157A grown in AMMO to O157A grown in SPENT (control) demonstrated that O157A growth in AMMO was significantly inhibited compared to O157A grown in SPENT at timepoints 4, 6 and 8 h (*p* < 0.05) (Figure 3C). In contrast, a comparison of OD enumerations for O157A grown in AMMO extracted from either TSB or TSB/EC to O157A grown in SPENT (extracted from same media as AMMO) did not have a significant difference in growth at timepoints 4, 6 and 8 h (*p* > 0.05) (Figure 3A,B, respectively). As expected, controls of O157A grown in either, fresh TSB, TSB/EC and EC had, in comparison, more cells than the corresponding spent media control (Figure 3A–C, respectively).

### 3.3. Effect of E. coli O103F Growth Stage on Molecule Production

**Figure 4:**
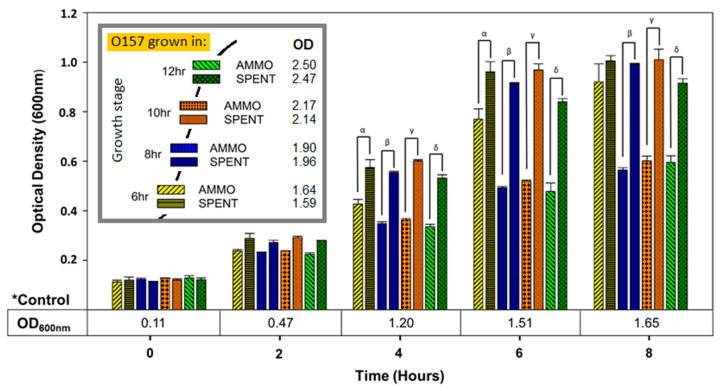
Time-time interaction. The effect of extraction time on ability of AMMO to inhibit growth over time of E. coli O157A measured using OD_600nm_ with AMMO extracted at 6, 8, 10 and 12 h. O157A control^(^*^)^ in fresh media is presented as a numerical value below the OD data. The symbols α, β, γ and δ denotes a significant difference between O157A grown in AMMO and in the SPENT for 6, 8, 10 and 12 h (*p* < 0.05). The recorded OD_600nm_ of AMMO and SPENT when extracted at each of the time points is shown in the legend. Note: (1) AMMO is the cell-free supernatant collected after 12 h of E. coli O103F growth. SPENT is the cell-free supernatant collected after 12 h of E. coli O157A growth. (2) Bars are the calculated standard deviation for the treated or untreated AMMO and SPENT at each time point.

Comparison of OD enumerations of O157A grown in AMMO extracted after 4 and 16 h of O103F growth to O157A grown in a matching SPENT demonstrated that AMMO did not inhibit growth of O157A (data not shown). In contrast, growth of O157A grown in AMMO extracted after 6, 8, 10, and 12 h was significantly inhibited (*p* < 0.05) compared to O157A grown in matching SPENT (Figure 4). The isolation time point of AMMO appears to affect inhibition parabolically, since the inhibitory effect grows larger over time up to the peak of 10 h and then declines (Figure 4). The control of O157A grown in fresh EC grew to a higher cell density than the corresponding spent media control (Figure 4).

### 3.4. Specificity of the E. coli O103F Molecule

**Figure 5:**
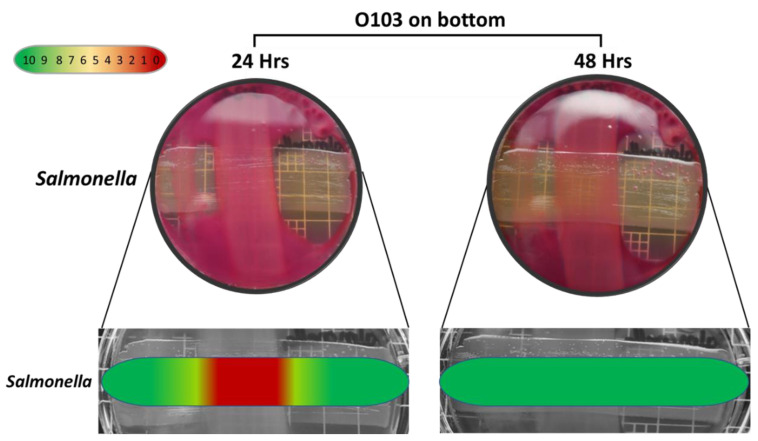
Omelette method results for *Escherichia coli* O103F against *Salmonella* on MAC at 24 and 48 h examining zones of no growth. Growth zones are graded beneath each plate with red (0) representing no growth to green (10) representing no inhibition of growth.

**Figure 6:**
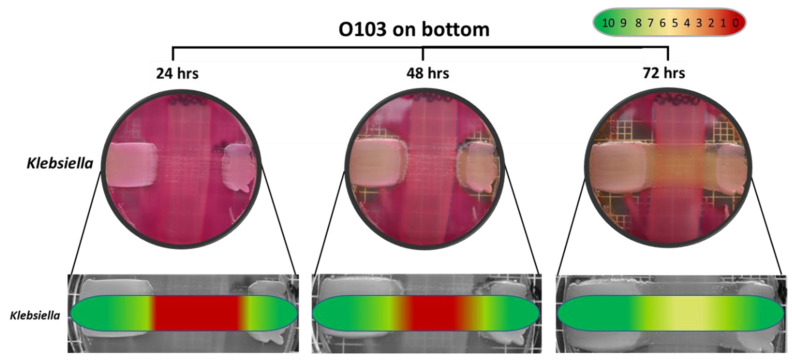
Omelette method results for *Escherichia coli* O103F against *Klebsiella* on MAC at 24, 48 and 72 h examining zones of no growth. Growth zones are graded beneath each plate with red (0) representing no growth to green (10) representing no inhibition of growth.

The semi-quantitative data examining the specificity of the O103F molecule identified that O103F molecule had an inhibitory effect on *Salmonella* at 24 h with a narrow zone of no growth, but the effect was lost at 48 h (Figure 5). In contrast, the O103F molecule had a stronger inhibitory effect against *Klebsiella* by maintaining a zone of no growth at both 24 and 48 h, but the effect diminished at 72 h (Figure 6).

## 4. Discussion

### 4.1. Effect of Carbon Source on Molecule Production

Previous results have revealed *E. coli* O103F as a strong competitor that produces a diffusible molecule(s) in EC liquid culture [20] and on MAC agar plates [19], capable of inhibiting the growth of 31 *E. coli* strains including STEC O157A. The same effect is seen in this trial with both EC and MAC, further confirming the previous results. However, when examining the effect of other media on molecule production it was noted that, while the *E. coli* strains thrived in all the media tested, the antimicrobial molecule was only produced and/or in an active stage when grown on/in EC and MAC. EC and MAC are similar in composition and, in comparison to TSB, the main differences are the presence of glucose (TSB), lactose (EC and MAC) and bile salts (EC and MAC), salts and potassium are comparable between all 3 media (Table S1, supplemental). Carbon source has been shown to affect gene expression in the presence of a prerequisite carbon source such as glucose, a phenomenon known as carbon catabolite repression (CCR) [24,25,26].

CCR, as a major regulatory mechanism, is thought to control up to 10% of all genes in bacteria [26]. The presence of glucose turns on CCR systems in *E. coli* and can affect many genes related to virulence or carbon utilization, as a study with *E. coli* O157:H7 found that adding glucose to LB lead to a significant reduction in indole and Shiga toxin synthesis at 37 °C [27], demonstrating the inhibitory effect of glucose. Furthermore, glucose has been shown to repress the production of some microcins [28,29]. The diffusible molecule produced by *E. coli* O103F is most likely a microcin [20]. Microcin production has been suggested to be under CCR regulation [30]. Possibly, glucose present in TSB media inhibited molecule production through CCR mechanisms. However, active antimicrobial molecule production was not observed when our selected strain was grown in LB, LBA, PN, MH, and TSA media, which do not contain any glucose, suggesting another mechanism triggers molecule synthesis.

Vice versa, it is equally feasible that molecule production is not modulated by glucose or other carbon sources but activated by the presence of lactose in EC, and MAC. *E. coli* has been shown to have different physiological properties when growing in lactose compared to glucose [24], and these differences could potentially affect the production of antimicrobial molecules. In the presence of lactose, *E. coli* growth is slower, cells are smaller and membrane protein content is changed to favour lactose degradation [24]. However, when lactose was added to TSB (TSB+), molecule production did not appear to be turned on since, *E. coli* O103F did not inhibit *E. coli* O157A growth. Overall, the results suggest that carbon source does not regulate molecule production in *E. coli* O103F and production is regulated by an alternative condition. Future projects examining selected media compositions may elucidate if individual or mixed carbon sources have an effect on antimicrobial molecule production for *E. coli* O103F.

Altogether, these results highlight an important point when examining the production of antimicrobials, such as microcins (in *E. coli* or other bacteria) the type of media and/or nutrients that appear to govern the ability of organisms to produce these molecules. A recent project screened *E. coli* isolates to identify bacteriocin producers using mitomycin C induction, but subsequent media used for agar-based competitions did not include the inducer, and most of the previously identified bacteriocin producers (using mitomycin C induction) did not inhibit their competitors [21].

Ultimately, the project design did not take into account the effect of media composition and the media used for screening differed from the media used in the agar-based competition assays. Another study examined the ability of non-pathogenic colicin-producing *E.coli* to inhibit *E.coli* O157:H7 but required the addition of mitomycin C to the LB media in order to induce colicin production [16], demonstrating that the media composition is of crucial importance when examining antimicrobial production. In addition, a study examining *E. coli* growth in LB determined that once the bacteria reach an OD_600nm_ of 0.3, they are no longer in steady state growth [31], microcin production is thought to be induced by stress conditions such as nutrient depletion [28], but the absence of observable antimicrobial activity in LB in our study provides evidence that antimicrobial production in *E. coli* O103F is governed by a specific elicitor. The results from this study and the above-mentioned research projects revealed that the type of nutrients can have an effect on antimicrobial production and should be considered when designing experiments to investigate antimicrobial molecules.

### 4.2. Effect of Bile on Molecule Production

Examining the effect of carbon source on antimicrobial production demonstrated that media containing lactose but no bile salts did not trigger the production in *E. coli* O103F, suggesting that bile salts may be responsible for activation. Examining the demonstrated effect of bile salts, a 50:50 mixture of TSB and EC containing a total of 750 ppm bile salts was sufficient to turn on molecule production in *E. coli* O103F, further confirming the above results: molecule production is not inhibited by TSB, but instead was turned on in the presence of bile. The composition of all media tested (liquid and agar) had only minor differences in salt and potassium and varied either in carbon source or by the addition of bile (Table S1, supplemental). Comparing all media used, only the presence of bile turned on the antimicrobial production. However, the results also revealed that decreasing the amount of bile salts resulted in the decreased activity of antimicrobial molecules in the TSB/EC mixture compared to EC. This was confirmed by a lack of significant inhibition (*p* > 0.05) of O157A growth in the mixture. Bile salts present in the small intestine of humans are considered bactericidal; a mechanism used to prevent overgrowth of bacteria and subsequently limit the loss of nutrients [11,22].

However, enteric bacteria such as *E. coli* have developed physiological adaptations to allow *E. coli* to either proliferate while in the presence of bile or to use bile as a cue to activate the stress response and turn on bile-counter mechanisms such as efflux pumps [4]. Bile has been shown to have an effect on the expression of various STEC O157:H7 genes, including LEE, flagella and bile resistance genes [13]. The study also identified that bile up-regulated genes associated with iron-acquisition, including genes related to the siderophore enterobactin, even in the presence of an iron-rich medium, suggesting that bile is an inherent cue for O157 to encounter a low-iron environment, regardless of the amount of iron actually present. The class IIb microcins, E492, H47 and M, are called siderophore microcins because the microcin is attached to a catecholate siderophore [32]. These microcins mimic catecholate siderophores and utilize siderophores receptors to gain entry into the competing bacterial cells, a Trojan horse [32,33]. Considering that the molecule produced by *E. coli* O103F is thought to be a microcin [20] and turned on by bile salts, which has been shown to turn on iron acquisition genes, suggests that O103F produces a siderophore microcin. Future research examining bile and gene expression may clarify the role of bile in turning on microcin production.

Outlook—a limiting factor of our study is the complex compositions of the commercial growth media used to examine the effect of individual components, such as pure carbohydrates or specific bile salt derivatives. Future studies with minimal media have the potential to further elucidate the role of single components on antimicrobial production.

### 4.3. Effect of E. coli O103F Growth Stage on Molecule Production

The examining growth stage on molecule production revealed that *E. coli* O103F produces the antimicrobial molecule while actively growing during the exponential phase. Previous studies reported that almost all microcins are produced when bacterial cells enter stationary phase; however, this is not true for microcin E492, which is produced in the exponential phase [28]. Microcin E492 is produced by *Klebsiella pneumoniae* and is a channel-forming bacteriocin [34]. Contrary to all other microcins described to date, the siderophore microcin E492 is only active in the exponential phase due to a lack of expression of genes needed during maturation in stationary phase. These results, along with the finding that the molecule produced by O103F is activated by bile salts, strongly suggests that O103F produces a microcin with properties similar to E492. Future projects examining the whole-genome sequence of *E. coli* O103F may elucidate similarities or differences in microcin gene clusters by comparing, among others, the coding sequences from E492 and O103F.

It has been proposed that not all bacteriocins possess the same role in producing cells, as some may be oriented as an offence or others as a defense mechanism [35]. Conceivably, a molecule produced in the exponential phase may not have the same role as a molecule produced in the stationary phase. Potentially, one may be characterized as an “attack molecule” pouncing before nutrient depletion to invade a new niche and another a “defense molecule” protecting what is left from competitors to ensure survival in stressful conditions. Further research may elucidate which impact the type of diffusible produced by *E. coli* O103F has in the cell and further clarify the roles these molecules have in microbial communities.

### 4.4. E. coli O103F Molecule Specificity

Microcins and colicins generally have a restricted killing spectrum, targeting closely related species and/or the same species [17,36]. More recently, they are being considered as replacements of medical antibiotics due to this unique trait. Our previous results have shown the diffusible molecule from *E. coli* O103F inhibits growth of various STEC and non-STEC strains monitored for up to 7 days [19], and the results from this study supplement that data by illustrating the diffusible antimicrobial also has inhibitory effects on *Salmonella* (24 h) and *Klebsiella* (48 h). The data from this study are in accordance with previous studies [17], since both species tested are closely related to *E. coli* and belong to the same family, Enterobacteriaceae [37]. Further research testing more diverse bacteria may further elucidate the range of bacteria which the *E. coli* O103F molecule targets.

The narrow target range of microcins, such as J25, is one of the features that make microcins an efficient option to target specific pathogens without affecting the remaining microbiome in the gut environment [38]. Traditional antibiotics kill many different bacteria indifferently at once, not only the target pathogen, which can lead to dysbiosis, an imbalance in the gut microbiome and various gut disorders [39]. Therapeutically important for the treatment of STEC infections is that traditional antibiotics are not recommended, due to the risk of activating toxin production [40], and alternate approaches are needed for medical interventions. Potentially, the type of antimicrobial molecule produced by *E. coli* O103F could be used as a therapeutic. Further research examining the effect of the molecule in animal models would be needed to measure the molecule effect on the whole gut microbiome to verify the targeted killing activity.

Bacteria, such as *E. coli* are constantly adapting and responding to their environments by evolving their physiological responses to external cues to ensure their survival [2,3]. *E. coli* habitually reside in the gastrointestinal tract of warm-blooded animals [5] and have adapted to these environments by responding to various cues, such as the presence of bile salts and carbon sources [4,7,26] and to turn on/off gene expression through mechanisms such as CCR [4,7,26]. Pathogens such as the STEC O157:H7 have been shown to respond to quorum-sensing molecules produced by the microbiome in the large intestine to regulate virulence genes [41] and are thought to have gained the ability to use sucrose due to the sucrose-rich diet of their host [42], further demonstrating how *E. coli* adapts to its ecosystem.

STEC such as O157:H7 are a significant food-borne pathogen [43], which are well adapted to their hosts [44], and current STEC mitigation strategies are not impeccable and often lack efficacy [45,46,47]. Producers of diffusible molecules, such as *E. coli* O103F, have shown promise to be used as an STEC mitigation strategy [16]. However, the presence of colicin and microcin genes in the genetic repertoire of an organism does not necessarily warrant the production of the antimicrobial, as shown here and in a previous study [21]; growth conditions do contribute to the production of diffusible molecules. Similarly, the microcins of the strong probiotic *E. coli* Nissle strain are only produced in iron-limiting media or conditions such as the inflamed gut [48], suggesting that the holistic mechanism and factors governing the microcin synthesis in the intestine are not yet completely understood. The type of molecule produced by *E. coli* O103F shows promise to potentially be used as a probiotic to mitigate pathogens like STEC. Bile salt concentrations appear to be a physiological cue along the GIT to turn on antimicrobial activity. Further investigation of regulatory mechanisms of the O103F antimicrobial activity may elucidate how to optimize the antimicrobial potential of this type of molecule.

## 5. Conclusions

Microbial infections amalgamated with antibiotic resistance are a growing global health concern. The over-use in livestock and human medicine has led to the emergence of pathogens that are resistant to treatment. There is an urgent need to identify alternative treatment approaches. Diffusible antimicrobial compounds are promising as potential agents to replace antibiotics. Previous work has shown that *E. coli* O103F, isolated from bovine feces, produces a strong antimicrobial compound that inhibited the growth of a wide range of virulent *E. coli* isolates. Our data reveal that the synthesis of the antimicrobial molecule is governed by the presence of bile salts, but not as assumed by different carbohydrate sources. We hypothesize our *E. coli* champion responds to bile as a physiological clue to turn on antimicrobial activity to compete for space and resources in the host intestine. Antimicrobials, such as microcins, have tremendous potential to mediate pathogens.

However, a better understanding of how these molecules are modulated by the host and the host microbiome is needed to optimize the inhibitory effects against target bacteria.

## Figures and Tables

**Table 1:** Commercial and modified media used in this study.

Media	Lab Code	Modification	Bile	Sugar	Supplier
*E. coli*	EC	none	1.5 g/L	lactose	1
Luria-Bertani (Miller)	LB	none	none		2
Luria-Bertani agar	LBA	none	none		2
MacConkey	MAC	none	1.5 g/L	lactose	2
Mueller-Hinton II	MH	none	none	starch	2
Nutrient agar	PN	none	none		3
Tryptic soy broth	TSB	none	none	glucose	1
Tryptic soy agar	TSA	none	none		1
Modified	TSB/EC	50:50 w:w	50% EC		
Modified	TSB+	5.0 g/L lactose	none		

1—EMD Millipore Canada; 2—BD USA; 3—Dalynn Canada.

## Supplementary Materials

The following are available online at https://www.mdpi.com/2306-7381/7/4/184/s1, Table S1: Composition of commercial and modified media used in this study, coupled with the bactericidal activity identified in these specific media, in this study, our previous studies [19,20] and by Cameron et al., [21] and Askari and Ghanbarpour [16], Figure S1: Comparison of cell numbers versus optical density after incubation in different media at time points 0, 4 and 8 h.

## References

[1] Biology Online: Physiological Adaptation. https://www.biology-online.com/dictionary/Physiological_adaptation.

[2] Pantin C.F.A. (1932). Physiological adaptation. Zool. J. Linn. Soc..

[3] Feugeas J.-P., Tourret J., Launay A., Bouvet O., Hoede C., Denamur E., Tenaillon O. (2016). Links between transcription, environmental adaptation and gene variability in *Escherichia coli*: Correlations between gene expression and gene variability reflect growth efficiencies. Mol. Biol. Evol..

[4] Sistrunk J.R., Nickerson K.P., Chanin R.B., Rasko D.A., Faherty C.S. (2016). Survival of the fittest: How bacterial pathogens utilize bile to enhance infection. Clin. Microbiol. Rev..

[5] Tenaillon O., Skurnik D., Picard B., Denamur E. (2010). The population genetics of commensal *Escherichia coli*. Nat. Rev. Microbiol..

[6] Jang J., Hur H.-G., Sadowsky M.J., Byappanahalli M.N., Yan T., Ishii S. (2017). Environmental *Escherichia coli*: Ecology and public health implications—A review. J. Appl. Microbiol..

[7] Luo C., Walk S.T., Gordon D.M., Feldgarden M., Tiedje J.M., Konstantinidis K.T. (2011). Genome sequencing of environmental *Escherichia coli* expands understanding of the ecology and speciation of the model bacterial species. Proc. Natl. Acad. Sci. USA.

[8] Conway T., Cohen P.S. (2015). Commensal and pathogenic *Escherichia coli* metabolism in the gut. Microbiol. Spectr..

[9] Nguyen Y., Sperandio V. (2012). Enterohemorrhagic *E. coli* (EHEC) pathogenesis. Front. Cell Infect. Microbiol..

[10] Hong W., Wu Y.E., Fu X., Chang Z. (2012). Chaperone-dependent mechanisms for acid resistance in enteric bacteria. Cell.

[11] Merritt M.E., Donaldson J.R. (2009). Effect of bile salts on the DNA and membrane integrity of enteric bacteria. J. Med. Micobiol..

[12] Cremers C.M., Knoefler D., Vitvitsky V., Banerjee R., Jakob U. (2014). Bile salts act as effective protein-unfolding agents and instigators of disulfide stress in vivo. Proc. Natl. Acad. Sci. USA.

[13] Hamner S., Mcinnerney K., Williamson K., Franklin M.J., Ford T.E. (2013). Bile salts affect expression of *Escherichia coli* O157:H7 genes for virulence and iron acquisition, and promote growth under iron limiting conditions. PLoS ONE..

[14] Hibbing M.E., Fuqua C., Parsek M.R., Peterson S.B. (2010). Bacterial competition: Surviving and thriving in the microbial jungle. Nat. Rev. Microbiol..

[15] Fabich A.J., Jones S.A., Chowdhury F.Z., Cernosek A., Anderson A., Smalley D., McHargue J.W., Hightower G.A., Smith J.T., Autieri S.M. (2008). Comparison of carbon nutrition for pathogenic and commensal *Escherichia coli* strains in the mouse intestine. Infect. Immun..

[16] Askari N., Ghanbarpour R. (2019). Molecular investigation of the colicinogenic *Escherichia coli* strains that are capable of inhibiting *E. coli* O157:H7 in vitro. BMC Vet. Res..

[17] Gillor O., Etzion A., Riley M.A. (2008). The dual role of bacteriocins as anti- and probiotics. Appl. Microbiol. Biotechnol..

[18] Riley M.A., Wertz J.E. (2002). Bacteriocins: Evolution, ecology, and application. Annu. Rev. Microbiol..

[19] Paquette S.-J., Zaheer R., Stanford K., Thomas J., Reuter T. (2018). Competition among *Escherichia coli* Strains for Space and Resources. Vet. Sci..

[20] Paquette S.-J., Reuter T. (2019). Properties of an antimicrobial molecule produced by an *Escherichia coli* champion. Antibiotics.

[21] Cameron A., Zaheer R., Adator E.H., Barbieri R., Reuter T., McAllister T.A. (2019). Bacteriocin occurrence and activity in *Escherichia coli* isolated from bovines and wastewater. Toxins.

[22] Urdaneta V., Casadesús J. (2017). Interactions between bacteria and bile salts in the gastrointestinal and hepatobiliary tracts. Front. Med..

[23] Njoroge J.W., Nguyen Y., Curtis M.M., Moreira C.G., Sperandio V. (2012). Virulence meets metabolism: Cra and KdpE gene regulation in enterohemorrhagic *Escherichia coli*. MBio.

[24] Kremling A., Geiselmann J., Ropers D., De Jong H. (2015). Understanding carbon catabolite repression in *Escherichia coli* using quantitative models. Trends Microbiol..

[25] Luo Y., Zhang T., Wu H. (2014). The transport and mediation mechanisms of the common sugars in *Escherichia coli*. Biotechnol. Adv..

[26] Görke B., Stülke J. (2008). Carbon catabolite repression in bacteria: Many ways to make the most out of nutrients. Nat. Rev. Microbiol..

[27] Medina M.B., Uknalis J., Tu S.-I. (2011). Effects of sugar addition in Luria Bertani (LB) media on *Escherichia coli* O157:H7. J. Food Saf..

[28] Duquesne S., Destoumieux-Garzon D., Peduzzi J., Rebuffat S. (2007). Microcins, gene-encoded antibacterial peptides from enterobacteria. Nat. Prod. Rep..

[29] Fang A., Demain A.L. (1997). Influence of aeration and carbon source on production of microcin B17 by *Escherichia coli* ZK650. Appl. Microbiol. Biotechnol..

[30] Salomón R.A., Farías R.N. (1992). Microcin 25, a novel antimicrobial peptide produced by *Escherichia coli*. J. Bacteriol..

[31] Sezonov G., Joseleau-Petit D., D’Ari R. (2007). *Escherichia coli* physiology in Luria-Bertani broth. J. Bacteriol..

[32] Vassiliadis G., Destoumieux-Garzón D., Peduzzi J., Drider D., Rebuffat S. (2011). Class II microcins. Prokaryotic Antimicrobial Peptides.

[33] Duquesne S., Petit V., Peduzzi J., Rebuffat S. (2007). Structural and functional diversity of microcins, gene-encoded antibacterial peptides from enterobacteria. J. Mol. Microbiol. Biotechnol..

[34] Corsini G., Baeza M., Monasterio O., Lagos R. (2002). The expression of genes involved in microcin maturation regulates the production of active microcin E492. Biochimie.

[35] García-Bayona L., Comstock L.E. (2018). Bacterial antagonism in host-associated microbial communities. Science.

[36] Kleanthous C. (2010). Swimming against the tide: Progress and challenges in our understanding of colicin translocation. Nat. Rev. Microbiol..

[37] Paterson D.L. (2006). Resistance in Gram-negative bacteria: Enterobacteriaceae. Am. J. Infect. Control.

[38] Naimi S., Zirah S., Hammami R., Fernandez B., Rebuffat S., Fliss I. (2018). Fate and biological activity of the antimicrobial lasso peptide microcin J25 under gastrointestinal tract conditions. Front. Microbiol..

[39] Francino M.P. (2016). Antibiotics and the human gut microbiome: Dysbioses and accumulation of resistances. Front. Microbiol..

[40] Pacheco A.R., Sperandio V. (2012). Shiga toxin in enterohemorrhagic *E. coli*: Regulation and novel anti-virulence strategies. Front. Cell Infect. Microbiol..

[41] Sperandio V., Mellies J.L., Nguyen W., Shin S., Kaper J.B. (1999). Quorum sensing controls expression of the type III secretion gene transcription and protein secretion in enterohemorrhagic and enteropathogenic *Escherichia coli*. Proc. Natl. Acad. Sci. USA.

[42] Moritz R.L., Welch R.A. (2006). The *Escherichia coli* argW-dsdCXA genetic island is highly variable, and *E. coli* K1 strains commonly possess two copies of dsdCXA. J. Clin. Microbiol..

[43] Smith J.L., Fratamico P.M., Gunther N.W., Sariaslani S., Gadd G.M. (2014). Chapter three—Shiga toxin-producing *Escherichia coli*. Advances in Applied Microbiology.

[44] Jubelin G., Desvaux M., Schüller S., Etienne-Mesmin L., Muniesa M., Blanquet-Diot S. (2018). Modulation of enterohaemorrhagic *Escherichia coli* survival and virulence in the human gastrointestinal tract. Microorganisms.

[45] Stanford K., Hannon S., Booker C.W., Jim G.K. (2014). Variable efficacy of a vaccine and direct-fed microbial for controlling *Escherichia coli* O157:H7 in feces and on hides of feedlot cattle. Foodborne Pathog. Dis..

[46] Stephens T.P., Stanford K., Rode L.M., Booker C.W., Vogstad A.R., Schunicht O.C., Jim G.K., Wildman B.K., Perrett T., McAllister T.A. (2010). Effect of a direct-fed microbial on animal performance, carcass characteristics and the shedding of *Escherichia coli* O157 by feedlot cattle. Ani. Feed Sci. Technol..

[47] Jin L., Wang Y., Iwaasa A.D., Li Y., Xu Z., Schellenberg M.P., Liu X.L., McAllister T.A., Stanford K. (2015). Purple Prairie Clover (*Dalea purpurea Vent*) reduces fecal shedding of *Escherichia coli* in pastured cattle. J. Food Prot..

[48] Sassone-Corsi M., Nuccio S.-P., Liu H., Hernandez D., Vu C.T., Takahashi A.A., Edwards R.A., Raffatellu M. (2016). Microcins mediate competition among Enterobacteriaceae in the inflamed gut. Nature.

