# Supplementary material for: Escherichia coli: Physiological Clues Which Turn On the Synthesis of Antimicrobial Molecules
Source: Vet Sci. 2020 Nov 21;7(4):184. doi: 10.3390/vetsci7040184 (PMC7712815; doi:10.3390/vetsci7040184)
Supplement: Supplementary file 1 [file vetsci-07-00184-s001.pdf]

Table 1. Composition of commercial and modified media used in this study, coupled with the bactericidal activity identified in these specific media, in this study, our previous studies [19, 20] and by Cameron et al, [21] and Askari and Ghanbarpour [16].

	Agar Media					Liquid Media				
	MAC	TSA	MH	PN	LBA	EC	TSB	LB	TSB+	TSB/EC 50/50 w/w
Composition in gram per Litre	Gelatin Peptone - 17	Casein - 15 (Pancreatic Digest)	Beef Heart Infusion - 2	Casein - 5 (Pancreatic Digest)	Tryptone - 10	Casein Digest - 20	Tryptone - 17 (Casein peptone)	Tryptone - 10	Tryptone - 17 (Casein peptone)	Tryptone - 8.5 (Casein peptone)
	Meat Peptone - 3	Soya Bean - 5 (Papaic Digest)	Casein Digest - 17.5	Beef Extract - 3	Yeast Extract - 10		Soy -3 (Soymeal Peptone)	Yeast Extract - 5	Soy -3 (Soymeal Peptone)	Soy -1.5 (Soymeal Peptone)
										Casein Digest - 10
	Lactose - 10		Starch - 1.5			Lactose - 5	Glucose - 2.5		Glucose - 2.5 Lactose - 5	Glucose - 1.25 Lactose - 2.5
	Bile Salts - 1.5					Bile Salts - 1.5				Bile Salts - 0.75
	Sodium Chloride - 5	Sodium Chloride - 5			Sodium Chloride - 10	Sodium Chloride - 5	Sodium Chloride - 5	Sodium Chloride - 10	Sodium Chloride - 5	Sodium Chloride - 5
	Agar - 13.5	Agar - 15	Agar - 17	Agar - 15	Agar - 15	Phosphate alloy - 4.5	Phosphate alloy - 2.5		Phosphate alloy - 2.5	Phosphate alloy - 4

Bactericidal activity identified in different media

	MAC	TSA	MH	PN	LBA	EC	TSB	LB	TSB+	TSB/EC
this study	+ +	−	−	−	−	+ +	−	−	−	+
Cameron [21]			−							
Paquette [19]	+ +					+ +				
Paquette [20]						+ +				
Askari [16]								−		

* Note: 1) Askari [19] used LB with mitomycin C to induce colicin production and for the purpose of this table, LB media without mitomycin C (as used in this study) is considered to not have induce bacteriocin production. 2) MAC also contains 0.03g of Neutral Red and 0.001g of Crystal Violet.

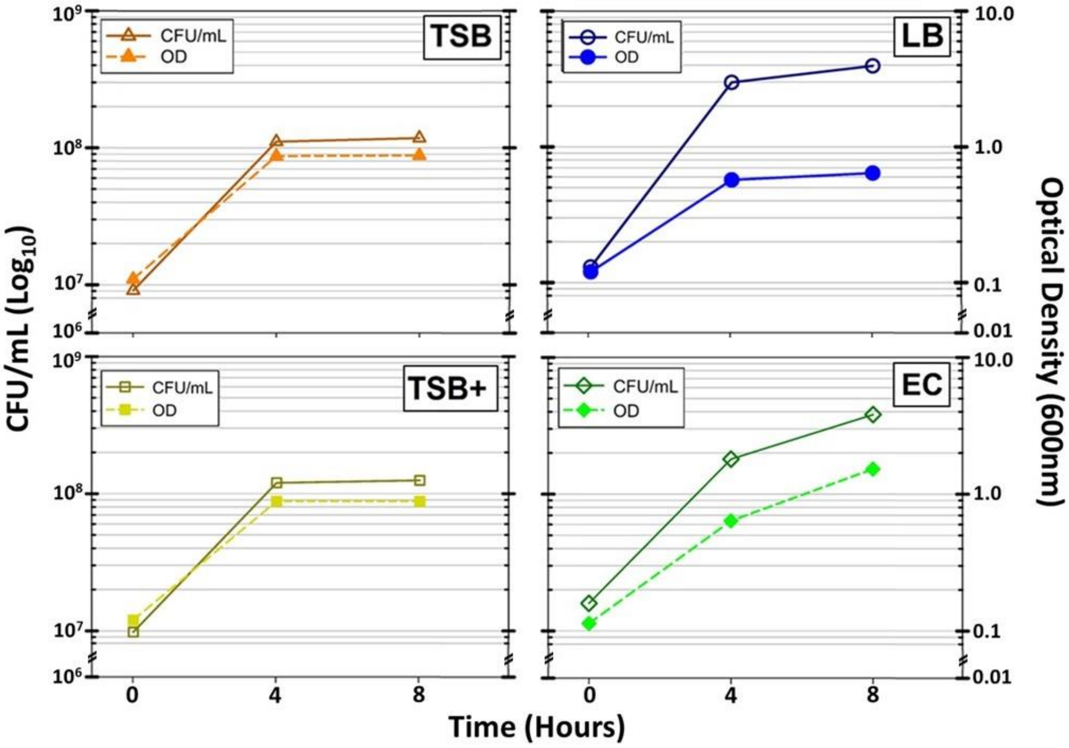

Supplemental Figure S1. Comparison of cell numbers versus optical density after incubation in different media at time points 0, 4 and 8 hours.
